# The Prevalence and Patterns of Maltreatment, Childhood Adversity, and Mental Health Disorders in an Australian Out-Of-Home Care Sample

**DOI:** 10.1177/10775595241246534

**Published:** 2024-04-16

**Authors:** Lottie G. Harris, Daryl J. Higgins, Megan L. Willis, David Lawrence, Ben Mathews, Hannah J. Thomas, Eva Malacova, Rosana Pacella, James G. Scott, David Finkelhor, Franziska Meinck, Holly E. Erskine, Divna M. Haslam

**Affiliations:** 1 School of Behavioural and Health Sciences, Institute of Child Protection Studies, 95359 Australian Catholic University, Melbourne, VIC, Australia; 2 Institute of Child Protection Studies, 95359 Australian Catholic University, Melbourne, VIC, Australia; 3 Faculty of Health Sciences, School of Behavioural and Health Sciences, 95359 Australian Catholic University, Sydney, NSW, Australia; 4 School of Population Health, 1649 Curtin University, Perth, WA, Australia; 5 School of Law, 1969 Queensland University of Technology, Brisbane, QLD, Australia; 6 Bloomberg School of Public Health, Johns Hopkins University, Baltimore, MD, USA; 7 QIMR Berghofer, Medical Research Institute, Brisbane, QLD, Australia; 8 Queensland Centre for Mental Health Research, Wacol, QLD, Australia; 9 Faculty of Medicine, 1974 The University of Queensland, Brisbane, QLD, Australia; 10 Institute for Lifecourse Development, 4918 University of Greenwich, London, UK; 11 Child Health Research Centre, 1974 The University of Queensland, Brisbane, Australia; 12 Child and Youth Mental Health Service, Children’s Health Qld, South Brisbane, QLD, Australia; 13 Crimes against Children Research Center, Department of Sociology, 3067 University of New Hampshire, Durham, NH, USA; 14 Faculty of Humanities, North-West University, Vanderbijlpark, South Africa; 15 School of Public Health, University of the Witwatersrand, Johannesburg, South Africa; 16 The Park Centre for Mental Health Treatment, Research and Education, Wacol, QLD, Australia; 17 School of Public Health, 1974 The University of Queensland, Brisbane, Australia; 18 Institute for Health Metrics and Evaluation, University of Washington, Seattle, USA; 19 Parenting and Family Support Centre, University of Queensland, Brisbane, QLD, Australia

**Keywords:** posttraumatic stress disorder, depression, population, foster care, child adversity, child maltreatment

## Abstract

This study aimed to explore key characteristics of the out-of-home care subgroup of a nationally representative Australian sample. To ensure that mental health services are appropriately targeted, it is critical that we understand the differential impacts of childhood experiences for this cohort. Using the Australian Child Maltreatment Study (*N* = 8503), we explored patterns of childhood maltreatment and adversity of participants who reported ever being placed in out-of-home care, such as foster care or kinship care. In addition, the prevalence of current and lifetime diagnosis of four mental health disorders were explored. Results showed that the care experienced subgroup reported more types of maltreatment and adverse experiences than the control group. They were also more likely to meet diagnostic threshold for post-traumatic stress disorder, generalised anxiety disorder and major depressive disorder than the control group. These findings can be used to guide mental health practitioners to target interventions more effectively within the out-of-home care cohort.

## Introduction

Accurate and reliable data that describe in detail the experiences that effect the wellbeing of children who enter statutory out-of-home care (OOHC) have the power to meaningfully impact the service system subsequently provided to them. At a national level in Australia, these data are sparse (Malvaso et al., 2020; Mathews et al., 2023). Across Australia, there is not one commonly accepted or adopted definition of child abuse or neglect, resulting in disparate collection and reporting of data on children and young people who come into contact with the child protection and out-of-home care systems (The Australian Institute of Health and Welfare, [AIHW], 2023). The Australian Institute of Health and Welfare collate and report national child protection data annually; however, concerns remain about the comparability of the data given that jurisdictions report on maltreatment notifications, investigations, and substantiations in significantly different ways (AIHW, 2023; McKenzie & Scott, 2011).

The incident-by-incident basis, on which maltreatment is formally reported also fails to adequately capture the breadth of historical and cumulative experiences of maltreatment that may not meet intervention threshold (Bromfield et al., 2007; Bryce, 2018). Even when children and young people have been the subject of substantiated reports of various types of maltreatment, often only the primary type of maltreatment is recorded (Runyan et al., 2005). In addition, data originating from statutory bodies such as child protection or health are known to underestimate the true prevalence of maltreatment (Austin et al., 2020; Kim et al., 2017), especially when compared to self-reported maltreatment experiences (Cooley & Jackson, 2022). The variation in reporting and data collection methods used across the child protection systems in Australia limit efforts to develop and implement national evidence-based strategies to tackle child maltreatment. Our ability to accurately investigate the impact that childhood maltreatment experiences have on later-in-life outcomes for all Australians has, historically, been hampered by our limited access to quality, detailed and comparable data on the maltreatment experiences themselves. Research and practice in the child protection and out-of-home care sectors rely on accurate maltreatment data to develop evidence-informed interventions that make a difference to children, young people, and their families.

The Australian Child Maltreatment Study (ACMS) responded to this data gap by producing rigorous, comparable, nationally representative evidence on the prevalence of maltreatment in Australia by addressing some of the foundational issues identified above. The ACMS data, collected in 2021, enable us to explore population-level maltreatment and adverse childhood experiences with greater detail than statutory reports allow, by utilising self-report mechanisms and definitionally sound and valid measures of maltreatment and adversity. Results of the ACMS show the alarming extent of maltreatment across the Australian population where 62.2% of the sample reported experiencing some form of maltreatment (Mathews et al., 2023), and almost two-thirds of these (39.4% of the population) reported more than one type of childhood maltreatment (multi-type maltreatment: Higgins et al., 2023). The study highlighted what many working in the child welfare sector already knew anecdotally, that child maltreatment is widespread and that experiencing a combination of maltreatment types is common. Substantial evidence has shown that poorer physical and mental health outcomes and increased psychopathology are associated with experiencing more than one type of childhood maltreatment more so than single type maltreatment (Bijlsma et al., 2023; Higgins & McCabe, 2000; Vizard et al., 2022). The polyvictimization research has also shown that an accumulation of both multiple maltreatment and adverse childhood victimization experiences has a particularly profound effect on wellbeing (Finkelhor et al., 2011; Mossige & Huang, 2017). Despite the extensive research on the effects of multiple maltreatment experiences, little research has provided details of these experiences for children and young people in OOHC anywhere in the world. The present study addresses this gap as the ACMS captured the childhood maltreatment and adversity experiences of people who reported an experience of living in OOHC.

The Australian Institute of Health and Welfare (AIHW, 2022b) reported that as of 30 June 2022, around 45,400 children (up to the age of 18 years) were placed in OOHC in Australia. Children and young people in OOHC live with a foster, or kinship carer or within a residential care setting, until they can safely return home, or they are able to live independently. Entering OOHC and the pursuant disconnection from family, culture, and community common for this group is considered itself, a compounding factor of maltreatment (Malvaso et al., 2022). Extensive literature in this area shows that the effects of childhood maltreatment contribute to life-long challenges, particularly on an individual’s mental health even after a short period of OOHC (Dubois-Comtois et al., 2021; Emmons et al., 2021; McLaughlin et al., 2021; Oh et al., 2018; Shmerling et al., 2020; Vizard et al., 2022).

Reliable and accurate data on the demographic characteristics and maltreatment histories of children and young people who enter statutory OOHC is essential to support the development and effectiveness of interventions targeted at this cohort. Although we are rapidly learning more about children and young people in OOHC in Australia, from comprehensive research studies such as the Pathways of Care Longitudinal Study (POCLS) and the New South Wales Child Development Study (NSW-CSD), there remains a gap in our knowledge in what happens to these children prior to their involvement with child protection services.

### The Present Study

The aim of this study is to explore the characteristics of the Australian Child Maltreatment Study (ACMS) subsample who reported having an OOHC experience. In particular, we are interested in their self-reported maltreatment and adverse childhood experiences as research has shown that using self-report measures offers a more accurate picture of maltreatment than do official, substantiated or un-substantiated reports (Mathews et al., 2020, 2023). We are also interested in key wellbeing indicators for this subgroup after their OOHC experience, namely their mental health diagnoses later-in-life. The overarching objective of this descriptive study is to generate a comprehensive overview of this cohorts’ experiences pre and post care, important to supporting public health approaches to prevention and response to child maltreatment for the group of children and young people who enter OOHC.

## Method

A subset of data from the ACMS was used in the present study. The ACMS applied a cross-sectional design, surveying a random sample of Australians by computer-assisted telephone interview. A representative population sample of 8503 Australians aged 16 years though to 65 years and older were asked to retrospectively recount their childhood experiences of maltreatment and other adverse childhood events. Questions related to their current and adult-life mental health concerns were also included in the survey. The Juvenile Victimisation Questionnaire (JVQ) – R2: Adapted Version (ACMS) was used to capture self-reported experiences of child maltreatment, a subset of the National Survey of Child Health items was used to assess adverse childhood events and the Mini International Neuropsychiatric Interview (MINI) was administered as a diagnostic measure of mental disorder, current and lifetime. The initial wave of the ACMS provides the first national benchmark data on child maltreatment experiences in Australia (Haslam et al., 2023). Further methodological details of the ACMS are available elsewhere (Haslam et al., 2023).

### Out-Of-Home Care

The ACMS included a singular question about OOHC experience as part of the set of questions about Adverse Childhood Experiences (ACEs), adapted from the original questionnaire (Felitti et al., 1998). This question was prefaced with the comment, “The next questions are about events that may have happened before you were 18” and continued “Were you ever placed in out-of-home care, such as foster care or kinship care?” Respondents had three answer options; Yes, No, I don’t know. They were also permitted to refuse to answer. In the current analysis, only those who answered ‘Yes’ were counted in the ‘care experienced’ subgroup and those who answered ‘No,’ or ‘I don’t know’ or who refused were categorised in the ‘not-care experienced’ subgroup.^1^

### Maltreatment and Adverse Childhood Experiences

Emotional abuse, sexual abuse, physical abuse, neglect, and exposure to domestic violence are the five types of maltreatment explored in the ACMS. Participants were regarded as having experienced maltreatment if they said yes to any of the screeners for physical abuse, sexual abuse, or exposure to domestic violence. They were deemed to have experienced emotional abuse or neglect if they reported that an experience occurred for longer than one week. Eight other non-maltreatment related ACEs items were included in the ACMS and were assessed using a subset of the National Survey of Child Health items. Participants were asked specifically whether, during their childhood, they had any of the following: lived with someone who had a mental illness, lived with someone who had a drug or alcohol problem, experienced unfair treatment due to their race, a parent or caregiver died, a parent/caregiver was incarcerated, parents or caregivers separated or divorced and whether they had ever been a victim of or witnessed violence. Each of these items used a binary scale, yes/no; however, the final item ‘experienced economic hardship’ used a categorical answer option. To reduce complexity of the model and increase power, we reclassified levels of childhood financial hardship into a binary variable. Specifically, we classified responses of ‘never’ or ‘not very often’ as no economic hardship and classified response options of ‘somewhat often’ and ‘very often’ as yes to experiencing economic hardship. Much like the OOHC question, the child maltreatment and adverse experiences questions were prefaced with instructions for participants to only answer for the period before turning 18 years old. Details of the rationale for including these five types of maltreatment and the eight ACEs, as well as the validation of maltreatment assessment tools are described in previous ACMS published reports (Haslam et al., 2023; Mathews et al., 2023).

### Mental Health

The ACMS survey assessed four major psychiatric disorders identified in the Diagnostic and Statistical Manual of Mental Disorders (DSM-IV); post-traumatic stress (current), generalised anxiety (current), alcohol use disorder (current), and major depressive disorder (lifetime) using the MINI, a validated and reliable brief psychiatric diagnostic tool. Alcohol use disorder (AUD) was separated into mild, moderate, and severe disorder categories consistent with the scoring criteria used in the DSM-IV and DSM-5.

## Data Analysis

Consistent with previous analyses from the ACMS, we conducted all analyses using data weighted by gender, age group, indigenous status, country of birth, highest educational level, and residential socio-economic status (Haslam et al., 2023; Mathews et al., 2023). For the present study, descriptive statistics were used to present prevalence estimates and 95% confidence intervals for exposure to maltreatment, the number of types of maltreatment, the combinations of maltreatment types experienced, other adverse childhood experiences, diagnosed mental health disorders and self-reported health risk behaviours. Our focus in this study was to explore these characteristics for the cohort that reported an OOHC experience. In so doing, we compared the maltreatment patterns for the care experienced and not-care experienced subgroups to determine whether groups differed on maltreatment patterns adopting the same approach as Higgins et al. (2023). Given that previous ACMS papers have reported these same prevalence estimates for the whole study sample (Higgins et al., 2023; Mathews et al., 2023; Scott et al., 2023), we occasionally refer to their data as a baseline from which to compare the care experienced subgroup while also comparing with the not-care experienced group. We also applied a logistic regression model to calculate the odds that participants would experience OOHC based on exposure to each of the eight adverse childhood experience risk factors, where the not-care experienced subgroup was used as comparison. We replicated analysis conducted in a previous ACMS paper (Scott et al., 2023) using odds ratios (ORs) and 95% confidence intervals, adjusting for age, gender and additionally, for this study, adjusted for multi-type maltreatment for care and not-care experienced subgroups.

The ACMS was approved by the Queensland University of Technology Human Research Ethics Committee (#1900000477). The Australian Catholic University Human Research Ethics Committee (ACU HREC) approved the analysis of a secondary data set (2023–3205N).

## Results

### Demographic Characteristics

Of the 8503 total ACMS participants, 395 (5.4% CI, 4.8–6.1%) had experienced a period of OOHC at some point during their childhood (0–18 yrs. old). The largest proportion of the care experienced subgroup (57.8%, *n* = 176, CI, 51.8–63.5%) were aged 45 years and older. Female respondents made up the majority of the care experienced subgroup at 58.4% (*n* = 213, CI, 52.4–64.2%). Almost 70% (*n* = 296, 69.3%, CI, 63.1–74.9%) of the care experienced subgroup were born in Australia while the rest were born overseas (*n* = 99, 30.7%, CI, 25.1–36.9%). The proportion of Australian-born respondents was slightly higher for those who also experienced OOHC compared to those who did not (*n* = 6051, 65.7%, CI, 64.3–67.1%). The supplemental material provides further demographic characteristics.

### Maltreatment Patterns

**Figure 1.**
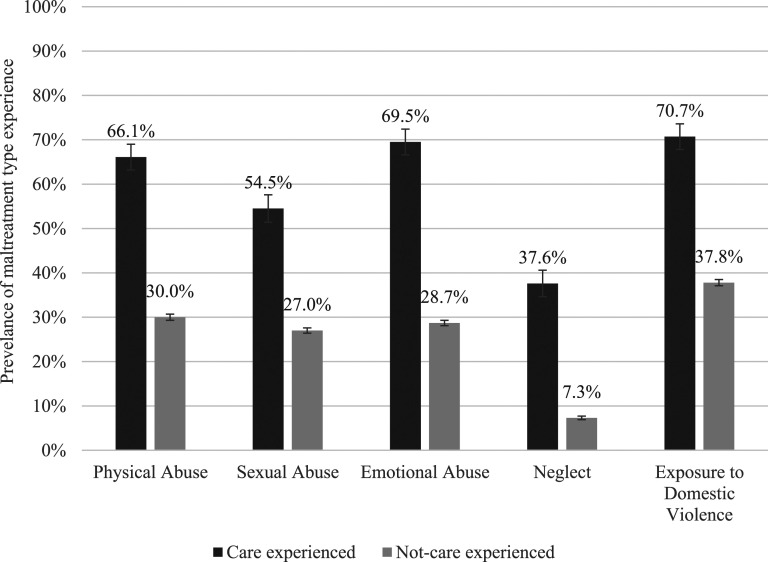
Weighted prevalence estimates (with 95% error bars) of maltreatment type experience by care and not-care experienced subgroups.

Nine in 10 of those in the care experienced subgroup self-reported having experienced one or more types of childhood maltreatment (*n* = 358, 91.5% CI, 87.6–94.3%) whereas only six in every 10 participants who had never been placed in OOHC reported experiencing any form of maltreatment (*n* = 4,922, 60.6% CI, 59.2–61.9%). Exposure to domestic violence was the maltreatment type most often endorsed by the care experienced subgroup at a prevalence rate of 70.7% (*n* = 288, CI, 64.7–76.1%), see Figure 1. Exposure to domestic violence was also reported with the greatest prevalence by the whole survey sample however at a much smaller proportion (39.6%; Higgins et al., 2023).

**Figure 2.**
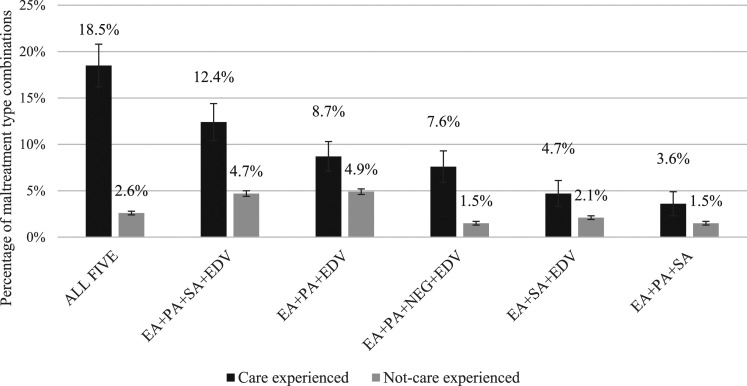
Weighted prevalence estimates (with 95% error bars) of the six most commonly reported combinations of multi-type maltreatment by care experience. *Note*. NB: EA: emotional abuse, SA: sexual abuse, PA: physical abuse, NEG: neglect, EDV: Exposure to domestic violence.

A critical finding of this study was that those within the care experienced subgroup reported higher rates of multi-type maltreatment (≥2 types of maltreatment; *n* = 311, 79.0%, CI 73.8–83.6%) than their not-care experienced peers (*n* = 3,053, 37.0%, CI, 35.8–38.5%), see Table 1. This pattern held true for each of the three age groups, see supplemental material for further details. When all age groups were combined, the least common maltreatment pattern for the care experienced subgroup was to have experienced only one type of maltreatment (*n* = 47, 12.4%, CI, 8.9–17.0%). Whereas the most common pattern was to report having experienced four types of maltreatment (*n* = 98, 25.0% CI, 20.1–30.6%). For the care experienced subgroup, the rate was progressively higher as the number of maltreatment types experienced was higher, which contrasts sharply with not-care experienced participants where the opposite pattern was observed, see Figure 2. The most common multi-type maltreatment combination for the care experienced subgroup was all five types together (*n* = 75, 18.5% CI, 14.3–23.4%). These results show that almost one in five children in OOHC will have experienced all types of maltreatment and more are likely to have experienced at least three. All multi-type maltreatment combinations reported by the OOHC subgroup included emotional abuse; and five of the six included exposure to domestic violence.

**Table 1.** Weighted Prevalence Estimates (With 95% CIs) of Multi-type Maltreatment by Care Experience (*N* = 8503).

	No Maltreatment *N*, % (95% CI)	Multi-type maltreatment (≥2 types) *N*, % (95%CI)	One type of maltreatment *N*, % (95% CI)	Two types of maltreatment *N*, % (95% CI)	Three types of maltreatment *N*, % (95% CI)	Four types of maltreatment *N*, % (95% CI)	Five types of maltreatment *N*, % (95% CI)
All Ages	3223; 37.8% (36.5–39.1%)	3378; 39.4% (38.1–40.7%)	1902; 22.8% (21.7–24.0%)	1342; 16.1% (15.1–17.1%)	1056; 11.7% (10.9–12.6%)	694; 8.1% (7.4–8.9%)	286; 3.5% (3.0–4.0%)
Care experienced	**37; 8.5% (5.7–12.4%)**	**311; 79.0% (73.8–83.6%)**	**47; 12.4% (8.9–17.0%)**	50; 13.2% (9.6–17.9%)	**88; 22.5% (17.8–28.0%)**	**98; 25.0% (20.1–30.6%)**	**75; 18.5% (14.3–23.4%)**
Not-care experienced	**3186; 39.4% (38.1–40.8%)**	**3067; 37.1% (35.8–38.5%)**	**1855; 23.4% (22.3–24.6%)**	1292; 16.3% (15.3–17.3%)	**968; 11.1% (10.3–12.0%)**	**596; 7.1% (6.5–7.9%)**	**211; 2.6% (2.2–3.1%)**

*Note*. NB: Confidence Intervals that do not overlap (indicating significant difference between groups) in bold.

### Adverse Childhood Experiences

**Figure 3.**
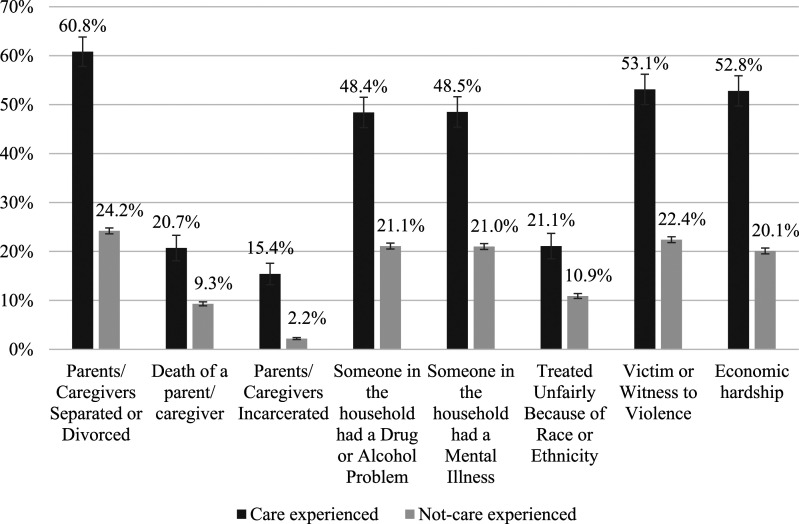
Weighted prevalence estimates (with 95% error bars) of experiencing Adverse Childhood Experiences by care experience.

The mean number of ACEs reported by the care experienced subgroup was 3.19 (*SD* = 1.12), whereas the mean number reported by the not-care experienced subgroup was 1.31 (*SD* = 0.02). The largest proportion of the not-care experienced subgroup (*n* = 3125, 38.0%, CI, 36.7–39.4%) did not endorse any adverse experiences during childhood; however, only a small proportion of the care experienced subgroup (*n* = 31, 7.6%, CI, 4.9–11.6%) reported the same. The care experienced subgroup reported all adverse childhood experiences at a higher proportion than their not-care experienced peers, at over twice the prevalence rate on each ACE item, see Figure 3. More than half of all survey respondents who had experienced OOHC reported that their parents had been separated or divorced, they had experienced economic hardship, and had been victim or witness to violence. Although death and incarceration of a parent/caregiver were endorsed by a much smaller proportion of both samples compared to other ACE items, the care experienced subgroup reported the death of a parent/caregiver at a prevalence rate two times that of the not-care experienced subgroup and reported incarceration of a parent/caregiver at six times the prevalence rate of the not-care experienced subgroup, see Figure 3.

Beyond the prevalence rates, we used survey weighted logistic regression to explore whether exposure to adverse childhood experiences increased the odds that a respondent would also experience OOHC. The results showed that experiencing each of the ACEs increased the odds of also reporting an OOHC experience, ranging from 1.54 (CI, 1.09–2.17) times for having an experience of being treated unfairly due to race or ethnicity to 4.43 (2.92–6.71) times for those with a parent/caregiver who was incarcerated, see Table 2. These analyses were adjusted for gender, age. We also adjusted for multi-type maltreatment given higher prevalence of multi-type maltreatment in the care experienced subgroup.

**Table 2.** Survey Weighted Logistic Regression for Out-Of-Home Care Experience in Australians 16 years and Older Who Experienced Adverse Childhood Experiences Relative to Those Who did Not.

Odds Ratio (95% CI) – adjusted for age, gender, and multi-type maltreatment	
Parents/caregivers separation or divorce	3.30 (2.48–4.41)
Death of a parent/caregiver	2.53 (1.79–3.59)
Incarceration of a parent/caregiver	4.43 (2.92–6.71)
Someone in the household had drug or alcohol problems	1.56 (1.18–2.07)
Someone in the household had a mental illness	1.68 (1.25–2.27)
Treated unfairly due to race or ethnicity	1.54 (1.09–2.17)
Victim or witness to violence	1.72 (1.29–2.30)
Economic hardship	2.07 (1.56–2.76)

### Mental Health Disorders

Results on the prevalence of mental health disorders show that 57.3% (*n* = 235, CI, 55.2–63.2%) of the care experienced subgroup had any mental disorder compared to 36.9% (*n* = 3,371, CI, 35.6–38.2%) of the not-care experienced subgroup, see Table 3. Of those who reported experiencing multi-type maltreatment during childhood, the care experienced subgroup maintained a higher prevalence of any mental disorders than the not-care experienced subgroup.

**Table 3.** Weighted Prevalence Estimates (With 95% CI) of Any Mental Disorder in Australians With and Without an Experience of OOHC, by Experience of Child Maltreatment (*N* = 8503).

Any Mental Disorder	Care experienced (*n* = 395)	Not-care experienced (*n* = 8108)
Total	**235; 57.3% (51.2–63.2%)**	**3371; 36.9% (35.6–38.2%)**
No maltreatment	7; 12.2% (4.7–27.9%)	844; 21.7% (20.0–23.5%)
Any maltreatment	**228; 61.5% (55.1–67.5%)**	**2527; 46.8% (45.0–48.6%)**
One type of maltreatment	16; 29.9% (17.3–46.6%)	748; 36.4% (33.7–39.2%)
Multi-type maltreatment (≥2 types)	**212; 66.5% (59.8–72.6%)**	**1779; 53.3% (51.1–55.6%)**
Two types of maltreatment	24; 48.1% (31.9–64.6%)	641; 44.4% (41.0–47.9%)
Three types of maltreatment	61; 66.2% (52.8–77.3%)	565; 55.5% (51.4–59.5%)
Four types of maltreatment	73; 75.4% (63.7–84.3%)	414; 65.1% (60.1–69.8%)
Five types of maltreatment	54; 67.8% (54.2–79.0%)	159; 67.7% (58.5–75.7%)

*Note*. NB: Confidence Intervals that do not overlap (indicating significant difference between groups) in bold.

Major Depressive Disorder (MDD) was also the most common mental health disorder present for the care experienced subgroup, which reported an even higher prevalence than the general population and not-care experienced subgroup at 30% (*n* = 123, CI 24.7–35.9%). It is important to note that prevalence estimates may have been affected by the fact that MDD was measured on a lifetime scale rather than using current diagnosis. The threshold criteria for Post-Traumatic Stress Disorder (PTSD) were met by 16.8% (*n* = 64, CI, 2.7–21.8%) of the care experienced subgroup, more than three times the proportion of the not-care experienced subgroup. The non-overlapping confidence intervals suggest a significant difference in the prevalence of PTSD diagnosis between these two groups, see Table 4. Mild Alcohol Use Disorder (AUD) was the only mental health disorder that the care experienced subgroup showed lower prevalence rates for than the not-care experienced subgroup, see Table 5. Though, for both moderate and severe AUD diagnoses, the care experienced subgroup prevalence rates were higher than for the other, suggesting that the care experienced subgroup experience mental health issues at the more severe end of the disorder spectrum.

**Table 4.** Weighted Prevalence Estimates (With 95% CI) of Post-traumatic Stress Disorder, Generalised Anxiety Disorder and Major Depressive Disorder in Australians With and Without an Experience of OOHC, by Experience of Child Maltreatment (*N* = 8503).

	Care experienced (*n* = 395)	Not-care experienced (*n* = 8108)
Post-traumatic stress disorder ⁌		
Total	**64; 16.8% (12.7–21.8%)**	**424; 4.7% (4.1–5.3%)**
No maltreatment	*	41; 1.3% (0.9–1.9%)
Any maltreatment	**64; 18.3% (13.9–23.7%)**	**383; 6.9% (6.1–7.8%)**
One type of maltreatment	*	52; 2.6% (1.8–3.8%)
Multi-type maltreatment (≥2 types)	**63; 20.8% (15.8–26.8%)**	**331; 9.6% (8.4–10.9%)**
Two types of maltreatment	6; 6.7% (1.9–20.5%)	74; 6.1% (4.6–8.1%)
Three types of maltreatment	**15; 18.7% (10.4–31.5%)**	**78; 6.9% (5.2–9.1%)**
Four types of maltreatment	21; 22.8% (14.2–34.7%)	115; 17.5% (14.1–21.6%)
Five types of maltreatment	21; 30.5% (19.7–44.1%)	64; 20.7 (15.1–27.7%)
Generalised anxiety disorder ⁌		
Total	**95; 24.6% (19.7–30.3%)**	**1053; 10.9% (10.1–11.8%)**
No maltreatment	*	173; 4.4% (3.6–5.3%)
Any maltreatment	**93; 26.5% (21.36–32.6%)**	**880; 15.2% (14.0–16.5%)**
One type of maltreatment	*	180; 8.2% (6.8–9.9%)
Multi-type maltreatment (≥2 types)	**91; 30.2% (24.2–36.9%)**	**700; 19.6% (17.9–21.4%)**
Two types of maltreatment	9; 19.2% (8.6–37.6%)	212; 14.0% (11.8–16.5%)
Three types of maltreatment	24; 28.7% (18.4–41.9%)	208; 18.2% (15.3–21.4%)
Four types of maltreatment	28; 32.5% (21.9–45.1%)	191; 29.3% (24.9–34.1%)
Five types of maltreatment	30; 36.7% (25.1–50.1%)	89; 34.7% (27.1–43.2%)
Major depressive disorder †		
Total	**123; 30.0% (24.7–35.9%)**	**1593; 17.7% (16.7–18.8%)**
No maltreatment	*	327; 8.1% (7.1–9.3%)
Any maltreatment	**120; 32.4% (26.7–38.6%)**	**1266; 24.0% (22.5–25.5%)**
One type of maltreatment	10; 20.8% (10.4–37.3%)	353; 17.1% (15.1–19.3%)
Multi-type maltreatment (≥2 types)	110; 34.3% (28.0-41.1%)	913; 28.3% (26.3–30.4%)
Two types of maltreatment	9; 17.7% (8.0–34.5%)	329; 23.2% (20.4–26.2%)
Three types of maltreatment	32; 34.7% (23.3–48.0%)	309; 30.0% (26.5–33.8%)
Four types of maltreatment	36; 36.3% (25.3–48.8%)	210; 36.0% (31.3–41.0%)
Five types of maltreatment	33; 43.0% (30.3–56.6%)	65; 31.9% (24.2–40.6%)

NB: ⁌ Current. † Lifetime. * Cells with *n* < 5 redacted. Confidence Intervals that do not overlap (indicating significant difference between groups) in bold.

**Table 5.** Weighted Prevalence Estimates (With 95% CI) of Alcohol Use Disorder in Australians With and Without an Experience of OOHC, by Experience of Child Maltreatment (*N* = 8503).

	Care experienced (*n* = 395)	Not-care experienced (*n* = 8108)
Alcohol use disorder – mild ⁌		
Total	39; 7.9% (5.4–11.4%)	1019; 10.8% (10.0–11.7%)
No maltreatment	*	343; 8.9% (7.8–10.2%)
Any maltreatment	37; 8.0% (5.4–11.7%)	676; 12.1% (11.0–13.3%)
One type of maltreatment	*	250; 11.8% (10.1–13.8%)
Multi-type maltreatment (≥2 types)	36; 9.1% (6.1–13.3%)	426; 12.2% (10.9–13.8%)
Two types of maltreatment	7; 10.3% (4.2–23.1%)	179; 12.7% (10.6–15.2%)
Three types of maltreatment	6; 6.5% (2.6–15.0%)	135; 13.2% (10.7–16.2%)
Four types of maltreatment	13; 9.9% (4.8–19.3%)	86; 10.7% (8.1–13.9%)
Five types of maltreatment	10; 10.4% (4.9–20.6%)	26; 9.5% (5.7–15.6%)
Alcohol use disorder – moderate ⁌		
Total	28; 6.2% (4.0–9.4%)	458; 4.6% (4.1–5.2%)
No maltreatment	*	111; 2.8% (2.2–3.7%)
Any maltreatment	27; 6.7% (4.3–10.2%)	347; 5.8% (5.1–6.7%)
One type of maltreatment	*	98; 4.3% (3.3–5.6%)
Multi-type maltreatment (≥2 types)	24; 7.0% (4.4–11.0%)	249; 6.8% (5.8–7.9%)
Two types of maltreatment	*	98; 5.9% (4.5–7.7%)
Three types of maltreatment	7; 6.3% (2.3–16.3%)	87; 7.6% (5.8–9.8%)
Four types of maltreatment	11; 11.4% (5.9–20.8%)	42; 7.2% (4.9–10.3%)
Five types of maltreatment	5; 6.3% (2.4–15.2%)	22; 7.6% (4.5–12.6%)
Alcohol use disorder – severe ⁌		
Total	33; 7.7% (5.0–11.6%)	363; 4.4% (3.8–5.0%)
No maltreatment	*	62; 2.0% (1.4–2.7%)
Any maltreatment	33; 8.4% (5.5–12.6%)	301; 5.9% (5.2–6.9%)
One type of maltreatment	3; 3.7% (0.9–15.0%)	79; 4.5% (3.4–5.9%)
Multi-type maltreatment (≥2 types)	30; 9.2% (5.9–14.0%)	222; 6.9% (5.8–8.2%)
Two types of maltreatment	*	65; 4.9% (3.6–6.6%)
Three types of maltreatment	13; 12.6% (6.3–23.6%)	60; 6.3% (4.6–8.6%)
Four types of maltreatment	12; 11.8% (5.8–22.7%)	67; 10.3% (7.6–13.8%)
Five types of maltreatment	*	30; 12.2% (7.4–19.4%)

*Note*. NB: ⁌ Current. * Cells with *n* < 5 redacted. Confidence Intervals that do not overlap (indicating significant difference between groups) in bold.

When both care and not-care experienced subgroups reported experiencing the same number of maltreatment types, and when both reported multi-type maltreatment (≥2 types), the care experienced subgroup fared consistently worse with higher prevalence rates for all but mild AUD, see Tables 4 and 5. The highest prevalence of any mental health disorder was for those who had experienced multi-type maltreatment and reported an OOHC experience. Although there is some overlap on prevalence estimates for mental health disorders by participants who reported having experienced ‘any maltreatment’, when we focus in on those who experienced multi-type maltreatment, the observed difference between those with and without a care experience becomes significant.

## Discussion

In the present study, we explored the patterns of maltreatment, adverse childhood experiences, and mental health outcomes for the care experienced subgroup of the ACMS survey sample to understand some key characteristics of their childhood and later-in-life experiences. The self-report nature of the ACMS data provides an important addition to currently collected and reported data from statutory bodies and adds a level of nuance that has not previously been achieved through other means. To our knowledge, this is the first exploration of population level of the maltreatment experiences of the Australian OOHC cohort and one of only a very few studies globally to present such data.

Results of our analysis show high prevalence rates of maltreatment in the care experienced cohort, at over 90%. This high rate is unsurprising given the nature of entry into OOHC is primarily due to experiences of significant harm. The small proportion of those within the care experienced subgroup reporting no maltreatment may be explained by several factors, including being removed as a preventative measure before harm occurred (i.e., at birth), having been orphaned, not perceiving maltreatment as such, or participant recall issues.

Few people with an OOHC experience reported only having experienced one type of maltreatment during childhood, while the most common maltreatment experience in this subgroup was to report having experienced four types. The proportion of the ACMS OOHC subgroup who reported multi-type maltreatment experience (79%) was markedly higher than that reported by a prominent Scottish study (Cusworth et al., 2019) in which 64% of their sample of looked after children aged five and above reported multi-type maltreatment. In addition, a US study of data collected using the national Child Abuse and Neglect Data System (NCANDS) on children and young people in OOHC also reported significantly lower rates of multi-type maltreatment (27%: Conn et al., 2013) compared to results from the present study. Comparisons between these studies should however be approached with caution as there were notable differences in the maltreatment types reported within each, for example, exposure to domestic violence was not accounted for as a maltreatment type in either the Scottish or the US study. The small number of studies that have comprehensively explored maltreatment experiences of children and young people in OOHC and the difficulty in comparing those few studies suggests this is an area for much needed further research. Although the results of our present study are not overly surprising, they add to a considerable gap in the literature on the prevalence of multi-type maltreatment in care experienced samples. These findings indicate that interventions that focus on single-type maltreatment experiences may not be suitable for the OOHC population; rather, holistic approaches that assume multiple types of maltreatment (even if documentation on maltreatment history is sparse) would be better suited.

These results, coupled with the high prevalence rates of mental health issues associated with multi-type maltreatment, build on previous research that has identified that children and young people in OOHC have high mental health needs (Green et al., 2020; Vasileva & Petermann, 2017) and that the greater number of maltreatment types experienced, the poorer the wellbeing outcomes typically (Bentovim et al., 2021; Vizard et al., 2022). This study has shown that in a population sample, the combination of experiencing both multi-type maltreatment and an OOHC placement increases the likelihood of poor mental health outcomes later in life beyond the impact that either one of those experiences has by itself. Our findings in this regard are similar to those produced by Lueger-Schuster et al. (2018) who reported that when matched on the most severe childhood maltreatment experiences, a sample of Austrian adults who had been in OOHC as a child experienced persistently poorer mental health outcomes later in life, compared to the control group who had not lived in OOHC. This suggests that the OOHC cohort are particularly vulnerable to mental health issues and a group that would greatly benefit from proactive mental health intervention.

Interestingly, emotional abuse appeared in all six of the most common combinations for the care experienced subgroup. This was slightly different to the whole survey sample where exposure to domestic violence was present in all of the most common multi-type maltreatment combinations and emotional abuse in only four combinations (Higgins et al., 2023). This should be considered an important finding for services working with children and young people in OOHC, given previous research that has demonstrated the impact emotional abuse can have (Lawrence et al., 2023; Spinazzola et al., 2014; Teicher & Samson, 2016). Importantly, Lawrence et al., found that the association between emotional abuse and poor mental health outcomes was as strong as for sexual abuse.

Beyond maltreatment experiences, we have shown that family-level factors including the separation, divorce, death and incarceration of a parent/caregiver are strongly associated with an OOHC experience. Our findings suggest that children and young people are at greater risk of child protection system surveillance and intervention when they experience these family-level adversities. Taking a public health approach, these experiences of family adversity should be seen as critical points at which targeted, intensive support could help families manage stressors in a manner that promotes child safety and wellbeing. Beyond adverse experiences that occurred within the family, community level adversities were also experienced by the OOHC subgroup at far higher rates than the not-care experienced group. Double the proportion of care-experienced respondents reported having been treated unfairly due to their race or ethnicity compared to not-care experienced respondents. Although we do not have further data to sufficiently explain these findings the significant over representation of Aboriginal and Torres Strait Islander children and young people currently and historically involved in the child protection system is likely one cause for this cohort to have experienced racism and unfair treatment at a higher rate. The prevalence of all ACEs in the OOHC cohort also suggest that including consideration of childhood experiences beyond maltreatment within mental health clinical assessments and practice may be of benefit. Ultimately, more information on the patterns of maltreatment and adverse experiences of children and young people that enter OOHC has the potential to inform referrals, services, and therapeutic programs (Collin-Vézina et al., 2011).

### Limitations and Future Research Directions

This study had several limitations, not least of which is the broad way in which OOHC was categorised within the ACMS. By only asking one question about the OOHC experience meant that data were limited on the context surrounding this experience, for example, placement type (i.e., foster, kinship or residential care), age of entry to care, length of time in care, and the number of placements. These factors are likely to play an important role on the mental health, health risk behaviours and general wellbeing of the current ACMS OOHC group as has been demonstrated with other studied OOHC groups (Asif et al., 2023; Hu et al., 2023). Therefore, a more nuanced exploration of the factors associated with care while in OOHC is required to make sound causal assumptions about OOHC and mental health outcomes. The OOHC group is typically heterogenous, and future research would benefit from including placement type, number of placements, length of care period and other OOHC-specific factors as covariates in any analyses. Further work in this area would also benefit from diving deeper into the maltreatment experiences by exploring the dimensions of maltreatment such as severity, duration, and age of onset. These dimensions of maltreatment may provide more detailed explanation about the experiences the care experienced subgroup had compared to their maltreated peers who did not have an OOHC experience. Finally, another limitation of this study is that we did not include any adult experiences, such as current interpersonal violence or current financial stress as confounders in our analysis, all of which may have played an influencing role on current mental health diagnoses.

Despite these limitations, the results of this study suggest that further research that explores the specific association of OOHC experience on mental health disorders is warranted. Matching groups on specific dimensions of the maltreatment experience, rather than just type as well as matching on OOHC specific factors would provide further detail on this pathway to adult mental health issues.

## Conclusion

Children and young people in OOHC are a group vulnerable to poor mental health outcomes by virtue of their maltreatment and adverse childhood experiences. The level of maltreatment and adverse experiences information is often not well documented by statutory bodies nor received by OOHC or mental health providers, causing a disconnect between the client’s experience and the services they receive. In addition, while studies measuring the prevalence of maltreatment among national or representative populations are growing, we found very few studies that reported on the maltreatment experiences of children and young people in OOHC, for whom, having this knowledge could be vital for their future health and wellbeing.

This study calls for child protection systems across the country to put more emphasis on identifying the whole maltreatment history, specifically the different types of maltreatment and adversities experienced, of children and young people in contact with the system and to share this information more widely and incorporating it into care planning and specific treatment plans to address mental health issues. To provide effective interventions, mental health and OOHC practitioners working with this group require a greater knowledge of their clients’ experiences and a strong understanding of the common maltreatment experiences of their target client group to be able to mitigate the associated mental health disorders.

## Supplemental Material

Supplemental Material - The Prevalence and Patterns of Maltreatment, Childhood Adversity, and Mental Health Disorders in an Australian Out-Of-Home Care Sample Supplemental Material for The Prevalence and Patterns of Maltreatment, Childhood Adversity, and Mental Health Disorders in an Australian Out-Of-Home Care Sample by Lottie G. Haris, Daryl J. Higgins, Megan Willis, David Lawrence, Ben Mathews, Hannah J. Thomas, Eva Malacova, Rosana Pacella, James G. Scott, David Finkelhor, Franziska Meinck, Holly E. Erskine, and Divna M. Haslamin Child Maltreatment

## Note

1. The original inclusion of study participants who responded “I don’t know” to the OOHC question was in keeping with the overall methodological approach taken by other researchers using this dataset. Additional analysis was conducted excluding this respondent group and found that it made little difference to the overall results. In most cases the removal adjusted results by 0.1% and did not change confidence intervals. Given the insignificant effect combined with a desire to include as much of the sample as possible, we believe that including the “I don’t know” responses in the analysis is a suitable approach.
