# Supplementary material for: The Prevalence and Patterns of Maltreatment, Childhood Adversity, and Mental Health Disorders in an Australian Out-Of-Home Care Sample
Source: Child Maltreat. 2024 Apr 16;30(1):42–54. doi: 10.1177/10775595241246534 (PMC11656622; doi:10.1177/10775595241246534)
Supplement: Supplemental Material - The Prevalence and Patterns of Maltreatment, Childhood Adversity, and Mental Health Disorders in an Australian Out-Of-Home Care Sample [file sj-pdf-1-cmx-10.1177_10775595241246534.pdf]

**Supplementary material**

*Weighted prevalence estimates (with 95% CIs) of demographic characteristics by care experience (N = 8,503)*

| | **Demographic Characteristics** | **Care experienced (*n* = 395)** | **Not-care experienced (*n* = 8,108)** |
|---|---|---|---|
| **Gender** | Female | 213; 58.4% (52.4-64.2%) | 3969; 50.5% (49.1-51.9%) |
| | Male | **173; 39.9% (34.2-45.9%)** | **4022; 48.6% (47.2-50.0%)** |
| | Non-binary/Other | 9; 1.7% (0.8-3.7%) | 117; 0.9% (1.7-1.2%) |
| **Age group** | 16-24 | **134; 10.4% (8.4-12.7%)** | **3366; 13.8% (13.2-14.4%)** |
| | 24-44 | 85; 31.8% (26.3-37.9%) | 1915; 35.4% (34.1-36.8%) |
| | 45+ | 176; 57.8% (51.8-63.5%) | 2827; 50.8% (49.4-52.2%) |
| **Country of Birth** | Australia | 296; 69.3% (63.1-74.9%) | 6051; 65.7% (64.3-67.1%) |
| | Overseas | 99; 30.7% (25.1-36.9%) | 2057; 34.3% (32.9-35.7%) |
| **State/ Territory in which the majority of childhood was spent *** | NSW | **109; 30.9% (25.5-36.8%)** | **2105; 23.1% (22.0-24.3%)** |
| | VIC | 81; 19.8% (15.5-25.0%) | 1748; 20.5% (19.4-21.6%) |
| | QLD | 81; 14.9% (11.5-19.2%) | 1775; 19.0% (18.0-20.1%) |
| | WA | 32; 7.6% (5.1-11.3%) | 537; 6.1% (5.5-6.8%) |
| | SA | 17; 3.9% (2.2-6.7%) | 488; 6.4% (5.7-7.1%) |
| | TAS | 11; 2.5% (1.3-5.0%) | 190; 2.6% (2.2-3.1%) |
| | NT | 5; 1.7% (0.6-4.7%) | 56; 0.5% (0.4-0.7%) |
| | ACT | 7; 0.9% (0.3-2.2%) | 102; 0.9% (0.7-1.2%) |

NB: Confidence Intervals that do not overlap (indicating significant difference between groups) in **bold.** NSW = New South Wales, VIC = Victoria, QLD = Queensland, WA = Western Australia, SA = South Australia, TAS = Tasmania, NT = Northern Territory, ACT = Australian Capital Territory. * Does not include those who responded ‘I don’t know’ or ‘Not applicable’ therefore data does not equal 100% of study sample.

*Weighted prevalence estimates (with 95% CIs) of multi-type maltreatment by OOHC experience, and by age cohort (N = 8,503)*

| | **No Maltreatment N, % (95% CI)** | **Multi-type maltreatment (≥ 2 types) N, % (95% CI)** | **One type of maltreatment N, % (95% CI)** | **Two types of maltreatment N, % (95% CI)** | **Three types of maltreatment N, % (95% CI)** | **Four types of maltreatment N, % (95% CI)** | **Five types of maltreatment N, % (95% CI)** |
|---|---|---|---|---|---|---|---|
| **16-24 Years** | | | | | | | |
| Care experienced | **14; 10.9% (6.2-18.5%)** | **104; 76.5% (67.6-83.5%)** | 16; 12.7% (7.7-20.2%) | 17; 11.3% (6.9-17.8%) | **29; 21.6% (14.5-30.9%)** | **35; 26.5% (19.2-35.4%)** | **23; 17.1% (11.3-25.1%)** |
| Not-care experienced | **1354; 40.0% (38.2-41.9%)** | **1296; 38.6% (36.8-40.5%)** | 716; 21.4% (19.8-23.0%) | 517; 15.0% (13.7-16.4%) | **425; 12.7% (11.5-14.1%)** | **250; 7.8% (6.8-8.9%)** | **104; 3.1% (2.5-3.8%)** |
| **25 - 44 Years** | | | | | | | |
| Care experienced | **2; 2.9% (0.7-11.1%)** | **77; 92.1% (84.3-96.2%)** | **6; 4.9% (2.1-11.0%)** | 15; 19.3% (11.6-30.4%) | 14; 16.5% (9.8-26.5%) | **25; 31.6% (21.9-43.2%)** | **23; 24.7% (16.4-35.3%)** |
| Not-care experienced | **680; 35.0% (32.7-37.4%)** | **777; 41.6% (39.1-44.0%)** | **458; 23.4% (21.4-25.6%)** | 337; 18.3% (16.4-20.3%) | 231; 12.0% (10.5-13.7%) | **153; 7.9% (6.6-9.2%)** | **56; 3.4% (2.6-4.5%)** |
| **≥45 Years** | | | | | | | |
| Care experienced | **21; 11.1% (6.9-17.3%)** | **130; 72.4% (64.4-79.2%)** | 25; 16.5% (11.1-23.9%) | 18; 10.1% (6.2-16.0%) | **45; 25.9% (19.2-34.0%)** | **38; 21.1% (15.1-28.5%)** | **29; 15.3% (10.4-21.8%)** |
| Not-care experienced | **1152; 42.5% (40.3-44.4%)** | **994; 33.6% (31.7-35.6%)** | 681; 24.0% (22.3-25.8%) | 438; 15.2% (13.8-16.8%) | **312; 10.0% (8.8-11.2%)** | **193; 6.5% (5.5-7.5%)** | **51; 2.0% (1.4-2.6%)** |

NB: Confidence Intervals that do not overlap (indicating significant difference between groups) in **bold.**
